# Brain‐Computer Interfaces Using Flexible Electronics: An a‐IGZO Front‐End for Active ECoG Electrodes

**DOI:** 10.1002/advs.202408576

**Published:** 2024-12-18

**Authors:** Kyle van Oosterhout, Ashley Chilundo, Mariana P. Branco, Erik J. Aarnoutse, Martijn Timmermans, Marco Fattori, Nick F. Ramsey, Eugenio Cantatore

**Affiliations:** ^1^ Department of Electrical Engineering Eindhoven University of Technology Eindhoven 5612AP The Netherlands; ^2^ UMC Utrecht Brain Center Department of Neurology and Neurosurgery University Medical Center Utrecht Utrecht 3584 CX The Netherlands

**Keywords:** a‐IGZO, analogue front‐end, brain‐computer interface

## Abstract

Brain‐computer interfaces (BCIs) are evolving toward higher electrode count and fully implantable solutions, which require extremely low power densities (<15mW cm^−2^). To achieve this target, and allow for a large and scalable number of channels, flexible electronics can be used as a multiplexing interface. This work introduces an active analog front‐end fabricated with amorphous Indium‐Gallium‐Zinx‐Oxide (a‐IGZO) Thin‐Film Transistors (TFTs) on foil capable of active matrix multiplexing. The circuit achieves only 70nV per sqrt(Hz) input referred noise, consuming 46µW, or 3.5mW cm^−2^. It demonstrates for the first time in literature a flexible front‐end with a noise efficiency factor comparable with Silicon solutions (NEF = 9.8), which is more than 10X lower compared to previously reported flexible front‐ends. These results have been achieved using a modified bootstrap‐load amplifier. The front end is tested by playing through it recordings obtained from a conventional BCI system. A gesture classification based on the flexible front‐end outputs achieves 94% accuracy. Using a flexible active front end can improve the state‐of‐the‐art in high channel count BCI systems by lowering the multiplexer noise and enabling larger areas of the brain to be monitored while reducing power density. Therefore, this work enables a new generation of high channel‐count active BCI electrode grids.

## Graphical abstract

Rigid Silicon‐CMOS Brain‐Computer Interface chips (BCIs) can create hotspots due to their high power‐density, making implantation difficult. Flexible electronics provides reduced power‐density and ultra‐thin form factor without compromising performance. This will enable electronics intimately connected to the human brain to, e.g., decode human hand movements, paving the way to the next generation of fully implantable and minimally invasive BCI systems.

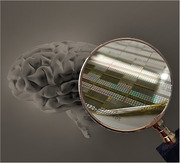

## 1 Introduction

NEUROLOGICAL conditions are becoming increasingly prevalent in an ageing society. Some of these conditions can cause the complete loss of motor control, leading to a condition described as locked‐in syndrome (LIS), which results in losing the ability to communicate.^[^ ^1^ ^]^ LIS is classically caused by vascular complications, such as a brainstem stroke,^[^ ^2^ ^]^ but is increasingly recognized in neurodegenerative disorders such as amyotrophic lateral sclerosis (ALS) in their later stages. After the onset of LIS, communication without external help is unlikely^[^ ^3^ ^]^ for a brainstem stroke and is completely lost in late‐stage ALS. To improve the quality of life significantly for individuals with LIS, Brain‐Computer Interfaces (BCIs)^[^ ^4^ ^]^ have been developed in the past, restoring communication ability in patients using non‐invasive electroencephalography (EEG).^[^ ^5^, ^6^, ^7^ ^]^ In recent years implantable systems have been developed that afford much higher reliability and performance.^[^ ^8^, ^9^, ^10^, ^11^ ^]^ Fully implantable BCIs have been developed for human use. Still, they are limited by the number of concurrent channels they can measure (up to 64 channels published to date), limiting the information that can be retrieved from the brain.^[^ ^9^ ^]^ Increasing the channel count could provide richer information from small patches of cortex^[^ ^12^ ^]^ and/or across larger areas of the brain, allowing to capture signals from multiple networked areas, and resulting in faster and more sophisticated gesture classification algorithms.^[^ ^13^ ^]^ This could enable individuals with LIS to communicate and live better.^[^ ^14^ ^]^ An increase in channel count will also inevitably cause a larger amount of interconnects to the data processing system (e.g., a Silicon analogue to digital converter), and therefore multiplexing is required at the electrode level.^[^ ^12^ ^]^ Indeed, when considering a wire‐bonded chip, which is the standard for mature process nodes (>14nm), the finest pad pitch that can be reached is 35µm.^[^ ^15^ ^]^ However, more standard pitches are between 50 and 100µm. This limits the number of external interconnects in a 1mm^2^ chip to roughly 80. Furthermore, the chip area needed to allow for the bond pad ring scales quadratically with the amount of pads. Thus, increasing the amount of connections to a Silicon integrated circuit is very cost‐ineffective. Besides, due to the large number of required bond pads, a large, rigid silicon chip is required. Using flexible electronics such as a‐IGZO, this challenge can be solved, distributing thin‐film transistors close to the brain on a large‐area conformable plastic substrate. Furthermore, the flexible solution has an ultra‐thin form factor (about 30µm thickness), which facilitates future implantation between the skull and the dura.

Previous works have already shown the benefit of flexible electronics in electrocorticography (ECoG) measurements,^[^ ^16^, ^17^ ^]^ demonstrating a state‐of‐the‐art 256‐channel system that achieves an input noise level of only 2.53µV_rms_ in a 500 Hz bandwidth. These works, however, used flexible electronics as a pure switching matrix, introducing the noise of the IGZO transistors in the system without providing gain or impedance transformation. Introducing a gain stage before the switching matrix could provide the same multiplexing benefits as shown in present literature, together with additional ones. The first improvement is that an anti‐aliasing filter can be implemented before the multiplexer, reducing the noise folding inherent to multiplexing. Indeed, the multiplexer needs to operate at a frequency of at least *f*_*mux*_ = 2*Nf*_*signal*_, where *f*_*signal*_ is the signal bandwidth, and N is the number of aggregated channels. Due to the signal components at the multiplexer frequency *f*_*mux*_, an anti‐aliasing filter after the multiplexer must be wide enough to pass at least *f*_*mux*_. Before the multiplexer a filter with a cut‐off frequency of *f*_*signal*_ can be used without loss of signal. Thus, filtering after, rather than before the multiplexer requires a 2*N* times higher bandwidth. The noise in this band will to be folded into baseband due to the sampling, and thus increases the noise voltage by at least $\sqrt{2N}$. To achieve anti‐aliasing before the multiplexer, an active element in the signal chain is required to filter out the frequencies above *f*_*signal*_, while providing low enough output impedance to allow the output of the multiplexer (and thus the sampler) to correctly settle within the required time window. Second, introducing an amplifier before the multiplexer can reduce the impedance seen by the connection wires, reducing the sensitivity of long interconnects to electromagnetic interferers, thus improving signal integrity. Furthermore, using large‐area electronics for the amplification stage spreads the power dissipation necessary to obtain the target noise floor over a much larger area, making it easier to keep the increase in temperature below the required 2°C.^[^ ^18^ ^]^ Multiple studies have worked at characterizing the maximum power consumption density that can be allowed in electronic systems implanted in the brain.^[^ ^19^, ^20^, ^21^ ^]^ Although the maximum power density changes depending on the exact application, these studies agree on a range between 15 and 130mW cm^−2^. As ECoG is similar to a multiple electrode array (MEA) without tips, as discussed in ref. [19], we assume in this work a maximum power density of 15mW cm^−^2. Such a low power density has only been approached on relatively large rigid silicon chips,^[^ ^22^, ^23^ ^]^ where 49 mW cm^−2^ has been achieved. When considering state‐of‐the‐art power consumptions per channel of 0.8µW,^[^ ^24^ ^]^ and an electrode count of 256 (the current ECoG state‐of‐the‐art), attaining this power density would still require a 1.37mm^2^ chip. Increasing the channel count would further increase the area needed. Indeed, even using alternatives to wire‐bonding, such as flip‐chip, Silicon circuits can create hot spots that are undesired for implant solutions in the brain.

There is thus a clear advantage of active analogue front‐ends (AFE) on flexible electronics for BCI applications. Such an AFE should have a maximum power density of 15mW cm^−2^, as explained in the previous paragraph. To be compatible with 5mm electrode grid spacing, the total area should be below 25mm^2^ per channel. Furthermore, the ECoG signal bandwidth of interest is between 70 and 125Hz,^[^ ^25^ ^]^ in which the signal level is between 0.3 and 1µV per $\sqrt{\mathrm{Hz}}$. Due to the robustness of the classification algorithm used, at least 0dB SNR is needed, which defines the maximum admissible noise level (see Section 4.1 for more information on the BCI specifications). In this work, we demonstrate a proof of concept for the use of a‐IGZO analogue front‐ends in BCI applications. This paper introduces a front‐end that amplifies the ECoG signals by 5V/V, while also providing the lowest input‐referred noise (IRN) and noise efficiency factor reported for an active TFT‐based front‐end to date. ECoG signals were recorded using an amplifier commonly used in clinical experiments for ECoG signal classification (Micromed SD128). These signals are provided to the input of the TFT front‐end. A gesture classification algorithm^[^ ^26^ ^]^ is applied to the resulting output, and reveals only a slight reduction (3%) in classification accuracy compared to the original signal due to the extra noise introduced by the AFE, thereby validating the TFT circuit functionality. The design explained here paves the road for a new generation of ECoG front‐ends by introducing a flexible AFE with a noise efficiency that is improved by more than 10X compared to previous state‐of‐the‐art, thus providing the option for high channel count, low noise flexible front‐ends that comply with the stringent power density limitations of implanted electronic systems.

## 2 Results

### 2.1 a‐IGZO Design Trade‐Offs

A major challenge in the design of AFEs using TFT technologies is the high level of 1/f noise. The design strategies used to limit the 1/f noise are usually to increase the size of the input transistors (up to a certain limit where the yield of the transistors starts dropping), and to decrease the current in the first stage. However, both these choices decrease the bandwidth of the circuit. Therefore, to design a circuit with a noise efficiency factor (NEF) approaching the one of silicon AFEs, the use of cancelation techniques for the correlated noise is required. Multiple such methods have already been exploited in flexible electronics, such as autozeroing,^[^ ^27^ ^]^ correlated double sampling (CDS),^[^ ^28^ ^]^ or chopping.^[^ ^29^ ^]^ Both autozeroing and CDS compare two sampled voltages (in analogue and digital domain respectively), increasing the white in‐band noise by $\sqrt{2}$, and requiring at least a twofold increase in sampling speed. Autozeroing additionally requires sampling at the amplifier level, introducing significant constraints on the area and noise trade‐off due to kT/C noise. Therefore, in this design, chopping is used to cancel most of the low‐frequency noise.

In chopper‐stabilized amplifiers, increasing the bias current *I*_*bias*_ of the amplifier will increase its bandwidth, allowing for higher chopping frequencies. However, the bias current will also increase the 1/f noise of the amplifier and decrease its thermal noise. Consequently, the noise corner frequency ‐ the frequency at which the 1/f noise power and the thermal noise power are equal—is moved to higher frequencies. Increasing the bias current for maximizing bandwidth thus comes at the cost of a higher noise corner frequency. Therefore, there is a need to derive the relationship between the equivalent input noise voltage and bias current. As a first‐order approximation for transistors working in the subthreshold regime, the transconductance scales linearly with the bias current. Therefore the gain‐bandwidth of the AFE,

(1) $${\mathrm{GBW}}_{AFE}=g_{m}/\left(2\pi C_{load}\right),$$

where *g*_*m*_ is the transconductance of the driver transistor, and *C*_*load*_ is the effective load capacitance of the amplifier, also scales linearly with the bias current. Assuming a constant gain *A*, we can write the bandwidth of the AFE as

(2) $${\mathrm{BW}}_{AFE}=KI_{bias},$$

where *K* is a constant. Because of the limited carrier mobility in large‐area technologies, it is usually not possible to design this bandwidth higher than the 1/f corner frequency, therefore we model the noise of the AFE as the 1/f noise of the transistor that acts as the dominant 1/f noise source. Assuming that the chopping frequency is equal to the bandwidth of the AFE, and thus that the in‐band noise after chopping is equal to 1/f noise at the corner frequency, the input‐referred noise becomes equal to^[^ ^30^ ^]^

(3) $$V_{n,rms(1/f)}=\sqrt{\int_{{\mathrm{BW}}_{AFE}-{\mathrm{BW}}_{signal}}^{{\mathrm{BW}}_{AFE}+{\mathrm{BW}}_{signal}}\frac{2\alpha_{H}q}{\gamma^{2}fW^{\frac{\gamma +1}{\gamma }}L^{\frac{\gamma -1}{\gamma }}C_{i}}\sqrt[{\gamma }]{\frac{I_{bias}}{\beta }}df},$$

where α_*H*_ is the Hooge factor,^[^ ^31^, ^32^ ^]^ *q* is the charge of an electron, *I*_*bias*_ is the bias current, *WL* is the area of the transistor, *C*_*i*_ is the capacitance per unit area of the technology and γ and β are technology parameters.^[^ ^33^ ^]^ Solving the integral, substituting (2), and normalizing the noise to the power consumption^[^ ^34^ ^]^ gives

(4) $$\begin{array}{ccc} & & \mathrm{NEF}\propto \sqrt{I_{bias}^{\frac{\gamma +1}{\gamma }}\ln\left(\frac{KI_{bias}+{\mathrm{BW}}_{signal}}{KI_{bias}-{\mathrm{BW}}_{signal}}\right)}\\ & = & \sqrt{I_{bias}^{\frac{\gamma +1}{\gamma }}\ln\left(\frac{BW_{AFE}+{\mathrm{BW}}_{signal}}{BW_{AFE}-{\mathrm{BW}}_{signal}}\right)}. \end{array}$$

The equation shows that, for AFE bandwidths larger than the signal bandwidth, the noise efficiency monotonically scales with *I*_*bias*_. Increasing the bias current thus increases (i.e., worsens) the NEF. Therefore, to obtain high efficiency, the bias current should be chosen as low as possible while remaining high enough to fulfil the noise (i.e., in‐band noise after chopping) requirements.

It is also important to size the input transistors well. Although the bandwidth and the noise power spectral density both scale in an inverse proportion to the area, the operating point of the transistor is important for the efficiency. It is optimal to choose a maximum transconductance (which is set by the noise requirements) for a given bias current. This maximum *g*_*m*_/*I*_*d*_ is found in the subthreshold regime, and therefore the width of the input transistor should be chosen accordingly, resulting in a large W/L.

### 2.2 Proposed AFE Architecture

Because a‐IGZO only provides n‐type transistors, conventional complementary amplifier architectures, such as inverter‐based amplifiers, are not available in this technology. Many circuit architectures have been proposed in literature to make unipolar amplifiers, such as resistive load,^[^ ^35^ ^]^ diode‐connected load,^[^ ^29^, ^36^ ^]^ positive feedback,^[^ ^30^, ^37^, ^38^, ^39^ ^]^ pseudo‐CMOS,^[^ ^40^, ^41^ ^]^ pseudo‐pmos,^[^ ^42^ ^]^ or bootstrapped load.^[^ ^43^ ^]^ In this work, a bootstrapped load amplifier is used for several reasons. First of all, due to the chopper, the input signal is upmodulated. Therefore, having a bandpass behavior for our amplifier is beneficial, so that low‐frequency errors such as offset are not amplified (which could introduce bias and linearity problems at the amplifier output, or in subsequent stages). Furthermore, bootstrapped load amplifiers can be designed with a trip point (where the amplifier's input voltage is equal to its output voltage) close to half the supply, allowing easy cascading of stages. Finally, the gain of a bootstrapped load amplifier can be designed close to *g*_*m*_ *r*_*o*_ without the use of positive feedback, which can be problematic in technologies that have large parameter variability, such as TFTs on flexible substrates.

**Figure 1:**
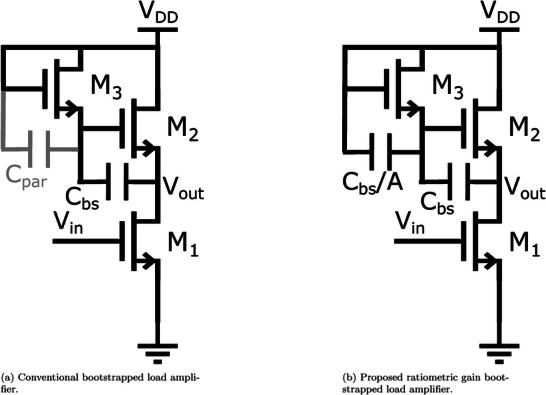
Bootstrapped load amplifier circuits.

The conventional bootstrapped load architecture is shown in **Figure** **1**a. To understand this architecture better, first, its low‐frequency behavior is analyzed. Low‐frequency in this case is defined as frequencies for which the capacitor *C*_*bs*_ can be considered an open circuit (1/(2π*fC*_*bs*_) > >*r*_*off*, *M*3_), where *r*_*off*, *M*3_ is the resistance of M3 in the off‐state (*V*_*d*_ = *V*_*g*_ = *V*_*s*_ = *V*_*DD*_). For these frequencies, the leakage current of *M*_3_ pulls up the gate of *M*_2_ to *V*_*DD*_. The reader could recognize this circuit as a diode‐connected load amplifier, which sets the bias point for the amplifier. At high frequencies (1/(2π*fC*_*bs*_) < <*r*_*off*, *M*3_), which is the frequency range of the signal, the bootstrap capacitor *C*_*bs*_ can no longer be considered as an open circuit. If we, for now, assume that the parasitic capacitance *C*_*par*_ is negligible, the impedance of *C*_*bs*_ will be much smaller than the impedance of the 0‐*V*_*ds*_ transistor *M*_3_ (*r*_*off*, *M*3_), and thus the gate of *M*_2_ will see the voltage *V*_*out*_, effectively shorting gate and source of *M*_2_ (*V*_*gs*_ = 0). This transistor, thus, offers a high impedance load at the signal frequencies, resulting in the desired high open‐loop gain. Unfortunately, the achievable in‐band gain in bootstrapped load amplifiers does not reach the theoretical limit of *g*_*m*, 1_ *r*_*o*, 2_, but is limited to a lower value, determined by the parasitic capacitance of *M*_2_ and *M*_3_, *C*_*par*_. This capacitance will create a voltage division between *C*_*bs*_ and *C*_*par*_, limiting the in‐band gain to

(5) $$\mathrm{Gain}=-\frac{g_{m,1}}{g_{m,2}}\frac{C_{par}+C_{bs}}{C_{par}},$$

where *g*_*m*, *X*_ is the transcondutance of transistor *X*. This equation is ratiometric. Therefore, setting *g*_*m*, 1_ = *g*_*m*, 2_ by sizing *M*_1_ = *M*_2_ and placing an explicit capacitor *C*_*bs*_/*A* between the gate of *M*_2_ and the gate of *M*_3_ which is much larger than the parasitic capacitance (Figure 1b), we can achieve a gain of

(6) Gain=−(1+A),

which is only dependent on the capacitor ratio under the assumption that *C*_*bs*_/*A* > >*C*_*par*_. This approach based on local feedback has the advantage of avoiding connecting the feedback network to the input nodes, resulting in a higher input impedance.

**Figure 2:**
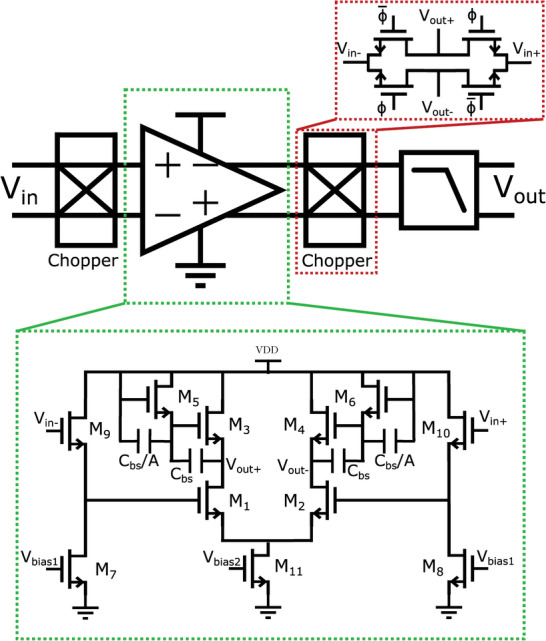
One channel of the proposed AFE, including a chopper‐stabilized amplifier using the proposed bootstrapped load architecture and an anti‐aliasing low pass filter.

Optimizing the bootstrap‐load amplifier variation discussed in this section taking into account the trade‐offs in a chopper circuit discussed above, results in a front‐end consuming 13µA and an input pair sized 3000x5µm, which places the corner frequency of the noise at around 16 kHz. The full single channel AFE, including choppers for noise reduction and a filter to prevent aliasing during the multiplexing and suppress the harmonics generated by the chopping is shown in **Figure** **2**. A buffer in the form of a level shifter is added before the amplifier to increase the input impedance of the AFE. Indeed, the parasitic capacitance between the gate and drain of *M*_1_ (*C*_*gd*, 1_) suffers from Miller effect, which increases the effective input capacitance by the gain of the amplifier.

The buffer is designed such that the power consumption and the noise are equal to those of the main amplifier, increasing the NEF by 2X.

### 2.3 Electrical Characterization

The front‐end circuit is designed and implemented using the unipolar a‐IGZO TFT technology provided by Pragmatic.^[^ ^44^ ^]^ This technology allows a supply voltage of 3.5V, a minimum feature size of 0.6µm. The TFT noise corner frequency lays at 20MHz for a minimum‐sized transistor with 1µA bias current.

**Figure 3:**
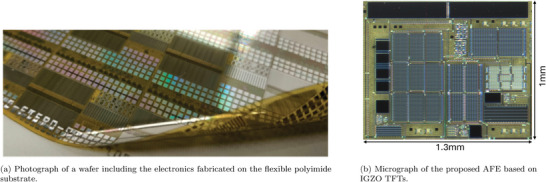
Images of the designed amplifier.

The proposed AFE circuit based on a‐IGZO TFTs is fabricated on a flexible polyimide substrate, occupies an area of 1.3mm^2^ per channel, and consumes a total power of 46µW from a 3.5V supply, resulting in a power density of 3.5mW cm^−2^. A photograph and the micrograph of the circuit are shown in **Figure** **3**a and Figure 3b respectively. In this section, the main measured performance indicators of the proposed AFE are provided.

**Figure 4:**
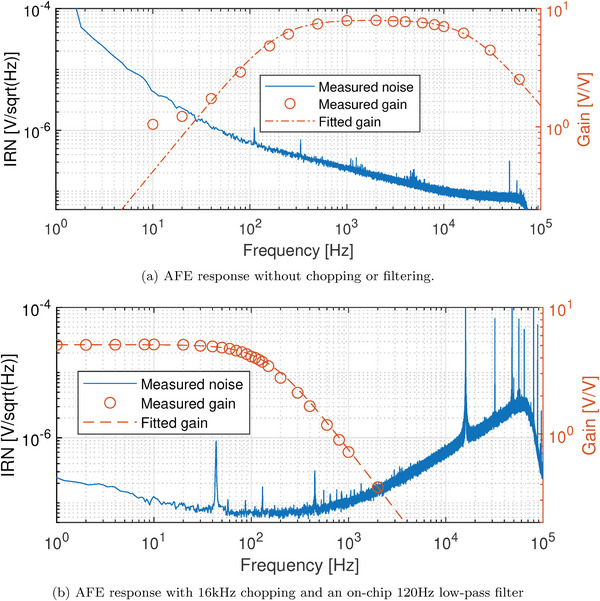
Measured gain (red) and input‐referred noise (IRN, in blue) performance of amplifier and full AFE. Peaks from the mains (50Hz and harmonics have been removed digitally).

**Figure** **4** shows results of the AC electrical measurements for both a channel consisting of only the amplifier without chopping (Figure 4a, red lines) and a channel consisting of the full AFE chopped at 16 kHz (chopper, amplifier, de‐chopper and filter) (Figure 4a, red lines). The amplifier without chopping shows a clear band‐pass behavior with a –3dB high‐pass corner around 200 Hz and a –3dB low‐pass corner around 20 kHz, indeed allowing for 16 kHz chopping. The total gain obtained is equal to 8V/V. When considering the chopped and filtered amplifier, the bandwidth is reduced to 120Hz, indicating the correct working of the filter. The total gain has however dropped to about 5V/V, which is a consequence of chopping close to the bandwidth of the system.

The measured Input‐Referred Noise (IRN) voltage spectral density is shown both with and without chopping in Figure 4a,b (blue lines). As expected, the results show clear 1/f behavior of the proposed amplifier (Figure 4a), with a noise corner frequency situated around 16kHz. Chopping and filtering brings the noise down to 70nV per $\sqrt{\mathrm{Hz}}$ in‐band, which is consistent with the noise level at 16kHz in the unchopped amplifier.

To confirm that the circuit would also work in a real application, at 70Hz, the CMRR is measured to be 67dB, the PSRR is measured to be 69dB, and the input impedance is measured to be 7.3MΩ. Measurements of the PSRR, CMRR and input impedance over frequency are shown in Figure S1 (Supporting Information). Even in the presence of ±150mV electrode DC offset (EDO), the performance of the amplifier remains within expectations, as shown in Figures S2 and S3 (Supporting Information). Furthermore, by adding an RC network (a capacitor of 1µF in series with a 1kΩ resistor) (Figure S4, Supporting Information) to electrically emulate each electrode, during the acquisition of a ECoG signal, the gain and the noise performance of the AFE circuit remain unaffected (Figure S5, Supporting Information). Finally, the circuit is simulated over PVT variations (Table S1, Supporting Information).

### 2.4 Validation Using Synthesized BCI Signals

**Figure 5:**
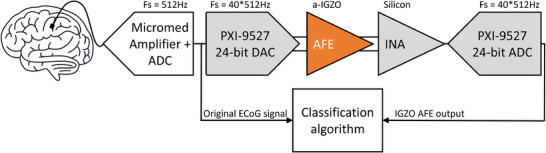
Block diagram of the measurement setup used for validation of the BCI system. Blocks in white are present in both the golden standard as well as the IGZO AFE test setup, while blocks in grey are only present in the IGZO AFE test setup. The orange block is the a‐IGZO AFE itself.

**Figure 6:**
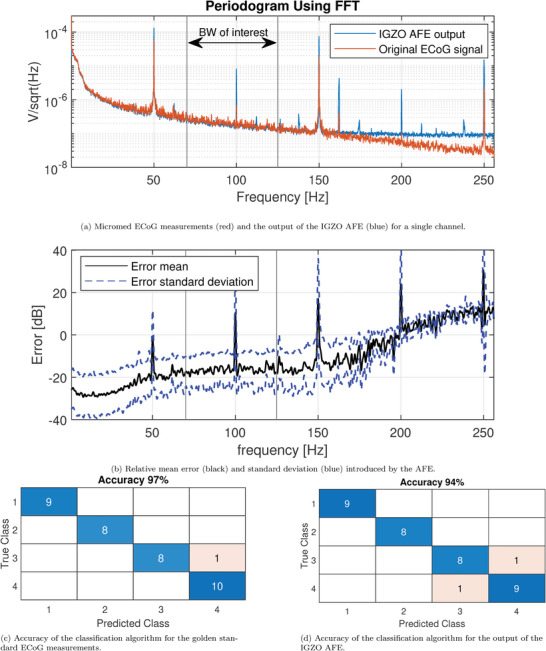
Results of the BCI system validation. In this figure the golden standard (red lines and the classification output in (c)) are shown as a reference, together with the output of the system when the a‐IGZO AFE is included in the processing chain (blue lines and the classification output in (d)).

Using the setup shown in **Figure** **5** (more information on the setup is given in Section 4.3), the output of the IGZO AFE is compared to the original ECoG data in **Figure** **6**. Figure 6a shows the FFT of the full 720‐s signal (the whole run) recorded from one representative channel for both the original ECoG data (red) and the IGZO AFE data (blue). At the frequencies of interest (<125 Hz), the measurements overlap, showing that the signal quality has indeed been preserved. For frequencies higher than 125 Hz, the signal level of the original ECoG data drops further, while the signal level of the AFE output flattens out. This occurs as the original ECoG signal falls below the AFE noise floor around this frequency range, leading to the signal being dominated by noise from these frequencies onward. These measurements have been repeated for 80 separate ECoG channels. The mean of the difference between the original ECoG data and the output of the IGZO AFE in the logarithmic domain as well as the standard deviation can be seen in Figure 6b (for more details on the error computation method, see Section 4.3). The figure shows that the mean error in the bandwidth of interest is about –20dB corresponding to an SNR of 20dB. Furthermore, the standard deviation of the error is only 10dB in the bandwidth of interest, and thus 95% of the channels remain within the noise limit of 0dB SNR.

**Figure 7:**
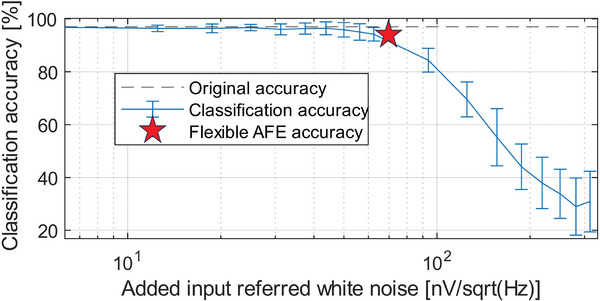
Accuracy of the classification algorithm with digitally added white noise assuming a gain of 5V/V.

The result of the gesture classification (see ref. [26] for more details on the classification algorithm) of both the original ECoG data and the AFE output can be seen in Figure 6c and Figure 6d respectively. The original ECoG data shows that one error out of the 36 included channels is made in the classification of the four hand gestures, while the AFE output shows two errors, indicating a slight degradation of the signal quality, lowering accuracy from 97% to 94%. These results are in line with the SNR computed for the 80 measured channels, as well as the expected signal degradation from the simulation study shown in **Figure** **7** (see Section 4.1 for more details).

## 3 Discussion

**Table** **1** compares the measurement results to the state of the art. This work is the first reported amplifier stage intended for ECoG‐based brain‐computer interfaces designed in flexible electronics. However, there have been previous works designing analogue front‐ends for biopotential readouts, such as EMG,^[^ ^27^ ^]^ ECG,^[^ ^29^ ^]^ EEG.^[^ ^35^ ^]^ Furthermore, the passive switching matrix of ref. [16] is also added for completeness.

**Table 1:** Comparison with state‐of‐the‐art flexible AFEs for biopotential readouts.

	Huang 2022^[^ ^17^ ^]^	Londoño 2023^[^ ^16^ ^]^	Moy 2017^[^ ^35^ ^]^	Zulqarnain 2020^[^ ^29^ ^]^	Genco 2023^[^ ^39^ ^]^	Van Oosterhout 2024^[^ ^27^ ^]^	This work
Technology type	Silicon	Flexible and Large‐Area Technologies					
Technology (feature size)	Si (22nm)	a‐IGZO (800nm)	a‐Si (6um)	a‐IGZO (1.5um)	a‐IGZO	a‐IGZO (800nm)	a‐IGZO (600nm)
Supply Voltage	0.8V	7V	55V	10V	26V	4V	3.5V
Architecture	I‐ΔΣ	Electrical switch	RL amplifier + 5kHz chopping	DCL amplifier + 1kHz chopping	PFL amplifier + 8kHz chopping	BSL amplifier + 10kHz Autozeroing	BSL amplifier + 16kHz chopping
Application	ECoG	ECoG	EEG	ECG	EMG	EMG	ECoG based BCI
Amplification	71dB	0dB	20dB	23.5dB	20µA/V	0dB	14dB
Noise level	70nV per sqrt(Hz)	120nV per sqrt(Hz)	230nV per sqrt(Hz)	800nV per sqrt(Hz)	2.46µV/sqrt(Hz)	2µV/sqrt(Hz)	70nV/sqrt(Hz)
Power dissipation/Channel	1.61µW	–	11mW	280µW	5.13mW	220µW	46µW
NEF	3.4	–	126	110	1338	1007	9.8
PEF	9.2	–	87.7e3	12e3	4.65e7	410e3	330
CMRR	98dB (N.A.)	–	50dB (100Hz)	67dB (50Hz)	76dB (N.A.)	–	67dB (70Hz)
PSRR	84dB (N.A.)	–	–	59dB (50Hz)	80dB (N.A.)	–	69dB (70Hz)
Input Impedance	43MΩ (N.A.)	–	260kΩ (DC)	16.5MΩ (50Hz)	25MΩ (N.A.)	250MΩ (50 Hz)	7.3MΩ (70Hz)
Area/Channel	1000µm^2^	900µm^2^	–	24mm^2^	16mm^2^	0.22mm^2^	1.3mm^2^
Power Consumption/Area	161mW cm^−^2	–	–	1.17mW cm^−2^	32.1mW/cm^2^	100mW/cm^2^	3.5mW/cm^2^

The main figure of merit that can be used to compare the different AFEs is the noise efficiency factor,^[^ ^34^, ^45^ ^]^ considering noise, current consumption, and bandwidth

(7) $$\mathrm{NEF}=V_{n,rms}\sqrt{\frac{2I_{tot}}{4\pi kTV_{t}\mathrm{BW}}},$$

where *V*_*n*, *rms*_ is the integrated input referred noise of the AFE, *I*_*tot*_ is the total current consumption of the AFE, and BW is its bandwidth. This equation does not, however, take into account the power supply, and therefore to take into account the actual power consumption, the power efficiency factor was introduced in ref. [46] as

(8) $$\mathrm{PEF}=\mathrm{VDD}\cdot {\mathrm{NEF}}^{2},$$

where VDD is the power supply of the AFE.

From the comparison table, we can conclude that the proposed amplifier has the lowest noise level, NEF and PEF published to date in the literature for large‐area electronics with 70nV per sqrt(Hz), 9.8 and 330 respectively. To understand why this work decreases the noise level so significantly compared to previous works, the easiest comparison would be to compare to a diode‐connected load amplifier such as reported in ref. [29]. Both structures use a limited number of transistors (2 for diode‐connected load, 3 for bootstrapped load). For a diode‐connected load amplifier, however, the gain is set by the ratio between the size of the driving transistor (*M*_1_) and the load transistor (*M*_2_). To achieve some gain, which is given by the first factor in Equation (2), and keep this gain accurate, the width of *M*_2_ is typically reduced to decrease its *g*_*m*_. This choice, however, increases the 1/f noise current generated by the load transistor, making it the dominant noise source. In this work, the load transistor is kept the same size as the driving transistor, and therefore the 1/f noise of the load does not dominate while the gain is kept accurate by the local capacitive feedback. Thus, for the same driving transistor size and biasing, the bootstrapped load amplifier proposed in this work can provide a ratiometric gain just like the diode‐connected load amplifier while reducing 1/f noise. Furthermore, this work has been designed in a more advanced technology node (600nm a‐IGZO), which enables increased TFT speed, allowing for higher chopping frequencies. The combination of improved circuit topology and technology allows this work to reach the best NEF reported in large‐area electronics to date and even reach a noise floor which competes with Silicon solutions.

It should be noted that the work^[^ ^17^ ^]^ does obtain a better NEF compared to this work, because that design is implemented in Silicon technology. Therefore, as expected, there is a clear trade‐off between using large‐area electronics only for passive multiplexing,^[^ ^16^, ^17^ ^]^ or implementing amplification too with large‐area TFTs (the approach used in this work). A more power‐efficient design can be obtained by designing the amplifier in Silicon technologies. The power consumption per area, however, will increase with this choice, as seen in the comparison table. Indeed, while the state‐of‐the‐art Silicon solution^[^ ^17^ ^]^ exhibits a power density of 161mW cm^−2^, large‐area solutions can reach power densities as low as 1.17mW cm^−2^. Therefore, most Silicon chips are unsuitable for implantation due to local heat dissipation and thus unsafe heating of tissue.^[^ ^19^ ^]^ Furthermore, including an amplifier and a suitable anti‐aliasing filter can reduce the noise folding inherent to multiplexing. Finally, only amplifying on the Silicon chip will require cabling from the large‐area electronics to the Silicon without amplification or impedance transformation, making it more prone to motion artefacts and electromagnetic interferences, while amplification of only 5 V/V on the flexible AFE can reduce the noise restraints of the Silicon chip by 25X, and thus reduce the power density by 25X, enabling implantable Silicon back‐ends.

Apart from the state‐of‐the‐art performance of the AFE, this work also demonstrates the feasibility of using large‐area electronics in ECoG biopotential readout systems, for example in BCI applications for individuals with locked‐in syndrome. In the future, this can enable a new generation of active electrode grids on flexible substrate with minimal wiring, which improve patient comfort and relaxed back‐end specifications, while ensuring the needed signal‐to‐noise ratio.

## 4 Experimental Section

#### BCI Specifications

For Brain‐Computer Interface (BCI) systems, it is often unclear what exact specifications are necessary to obtain a good classification output because there is no standardized way to decode ECoG signals. Some common specifications are the input‐referred noise (IRN), power consumption and bandwidth; however, all these requirements are highly dependent on the exact application and classification algorithm used to interpret the data. The approach used in this work, based on data acquired with the Micromed SD128 and a classification algorithm for hand gestures,^[^ ^26^ ^]^ will instead serve as a practical validation of the system where there are no clear, widely accepted specifications available.

Figure 7 shows the mean and standard deviation of the classification accuracy obtained when different amounts of thermal noise is added to the measured ECoG data, assuming the AFE has a gain of 5V/V. The figure shows that from about 60nV per $\sqrt{\mathrm{Hz}}$ input‐referred added noise, the accuracy of the classification starts to degrade. For this reason, the input‐referred noise for the BCI described in this work is specified to stay below this level. The bandwidth of interest in the classification algorithm is from 70 Hz to 125 Hz, which is chosen because the Micromed SD128 has an internal low‐pass filter at 134.4 Hz. For these specifications, the power consumption was to be minimized, such that the heat dissipation on the foil would also be minimized.

#### Classification Algorithm and ECoG Data for Validation

The feasibility of deploying the proposed AFE in an ECoG‐based system was tested by comparing the classification results based on the outputs of our AFE against data acquired by the golden standard system which UMC Utrecht used to obtain the ECoG signals: a commercial Micromed SD128 amplifier and ADC (128 channels, 22‐bit ADC, 0.15 Hz‐134.4 Hz bandpass system) sampled at 512 Hz, recording from the sensorimotor cortex hand region. Subjects of the study were five patients (mean age 31, range 19–45) with intractable epilepsy who were implanted with subdural ECoG grids to localize the seizure focus. This study was approved by the Medical Ethical Committee of the Utrecht University Medical Center. All patients signed informed consent according to the Declaration of Helsinki (2008), of which the data of one patient (subject 3) was chosen for this study based on the data quality. In the same way as in ref. [26], it is assumed here that the results of this work, that were obtained on patients affected by intractable epilepsy, can be extended to paralyzed patients. The participant was asked to make 4 complex hand movements (chosen from the letters “D,” “F,” “V,” and “Y” of the American sign language fingerspelling alphabet) during a 6‐s trial. Each gesture was repeated 10 times in random order, and interleaved with 6s rest trials. The recorded ECoG data was then classified using a high‐frequencyband power trace feature extraction method (for more details, please refer to^[^ ^26^ ^]^).

#### Electrical Characterization Setup

The electrical measurements were done by placing the designed a‐IGZO foil as an additional interface between the respective acquisition and classification blocks observed in a BCI system. Figure 5 shows this measurement setup as a block diagram. To make sure the signal‐to‐noise ratio would not degrade by returning the digitalized ECoG data to the analogue domain, a 24‐bit DAC (PXI‐9527) was used. Because this DAC is implemented with a Delta‐Sigma modulator, the data has to be oversampled to generate a high‐fidelity output. This was done by sending each ECoG data point forty times at a forty times higher speed (20.480 Hz). The same is true for the ADC, which was implemented using the same PXI‐9527 with equal resolution and sampling speed. The accuracy of the data conversion was tested by running the data of a single ECoG channel (channel 1) through the DAC and ADC, with no IGZO AFE interposed. No degradation of the SNR was observed during this trial.

AC measurements were performed utilizing a differential signal generator (Stanford ds360) to generate a 50mV_pp_ input signal on top of a 1.5V bias at various frequencies between 1 Hz and 60 kHz. The output was acquired using a PXI‐9527 24‐bit Sigma‐Delta ADC. This was done for both a channel consisting of only the amplifier (see Figure 4a, red lines) and a channel consisting of the full AFE system chopped at 16 kHz (chopper, amplifier, dechopper and filter) (Figure 4b, red lines). Noise measurements were performed using the same setup as the AC measurements, while setting the input signal to 0V (blue lines in Figure 4a and Figure 4b).

The accuracy of the BCI system in combination with the proposed a‐IGZO AFE was evaluated by running the signals from the 80 channels relevant to the classification through the measurement setup (Figure 5). Because the current implementation consists of only one channel, the channels were run in succession through the same amplifier. Because the DAC and ADC introduce some delay into the system, before the classification a pre‐processing was done to ensure the labels of the trials were still in the correct position. This was done by performing a cross‐correlation in Matlab between the original ECoG data and the newly acquired data. The acquired data was then time‐shifted such that the cross‐correlation was maximized at t = 0. The data was then classified using the same algorithm as Ref [26], and compared to the results of the classification of the original ECoG data.

To obtain Figure 6a, first the original ECoG signal for a single channel was split into 14.4‐s batches, for each of which an FFT was taken with a blackman window to reduce spectral leakage of the power line distortion. The average of the FFT values is plotted (red line). Afterwards, the same is done for the signal that has passed through the IGZO AFE (blue line). This process was repeated for all 80 channels, except for an extra gain correction step, which was performed by normalizing the signal to get the same power within a bandwidth of 20–30 Hz. The frequency spectrum of the error is computed using the function

(9) $$\mathrm{Error}=20\log_{10}\left(\frac{V_{AFE}-V_{ECoG}}{V_{ECoG}}\right),$$

where *V*_*AFE*_ is the voltage spectrum of the signal passed through the IGZO AFE, and *V*_*ECoG*_ is the voltage spectrum of original data.

To clearly show the trend of the error, a digital moving‐average filter with a filter size of 5.12 Hz was applied to the mean and standard deviation of the error function, resulting in the black and blue lines for the mean error and the standard deviation of the error respectively (Figure 6b).

## Conflict of Interest

The authors declare no conflict of interest.

## Author Contributions

The overall research was designed and supervised by E.C. and N.F.R.; the circuit design was carried out by K.O.; circuit design reviews were done by K.O., M.T., M.F., and E.C.; the classification algorithm was made by M.P.B.; the micromed ECoG measurements were performed by E.J.A.; Electrical characterization of the electronics was done by K.O.; Noise specification simulations were performed by A.C.; Validation of the electronics using synthesized BCI signals has been performed by K.O., and A.C.; K.O. wrote the first draft of the manuscript; editing and revision were carried out by K.O., A.C., E.J.A., M.T., M.F., N.F.R., and E.C.

## Supporting information

Supporting Information

## Data Availability

The data that support the findings of this study are available from the corresponding author upon reasonable request.
