# Supplementary material for: Brain‐Computer Interfaces Using Flexible Electronics: An a‐IGZO Front‐End for Active ECoG Electrodes
Source: Adv Sci (Weinh). 2024 Dec 18;12(6):2408576. doi: 10.1002/advs.202408576 (PMC11809407; doi:10.1002/advs.202408576)
Supplement: Supplementary file 1 — Supporting Information [file ADVS-12-2408576-s001.docx]

1. **Supporting Information**

**S.1 Process-Temperature-Voltage (PVT) simulations**

Using the PDK provided by Pragmatic, simulations were performed to evaluate the robustness of the circuit to changes in the process, temperature and supply voltage. The results of these simulations are summarized in TABLE S1. For the simulations including the electrode (“w/ electrode”), an electrode model (Z_elec_) consisting of a 1µF capacitor in parallel with a 1MΩ resistor, and a series resistance of 1kΩ is added between the voltage source and the input of the AFE. The bandwidth and gain are computed using AC analysis with the 120Hz output filter turned off (Figure 2). The transient noise is calculated using a transient simulation of 1.2 seconds (as a trade-off between simulation time and accuracy) and is integrated from 70Hz to 125Hz (the bandwidth of interest).

TABLE S1: PVT SIMULATION RESULTS OF THE AFE.

| PVT | Fast | Slow | Typical | 0°C | 85°C | VDD=3.3 | VDD=2.7 |
| --- | --- | --- | --- | --- | --- | --- | --- |
| Gain [V/V] | 6.305 | 4.561 | 6.138 | 4.017 | 6.663 | 6.145 | 6.129 |
| Gain [V/V]  w/ electrode | 6.274 | 4.551 | 6.122 | 3.958 | 6.656 | 6.128 | 6.114 |
| BW [kHz] | 131.5 | 16.33 | 107.6 | 13.39 | 199.1 | 107.4 | 107.8 |
| Tran noise IRN [nVrms] | 120 | 355 | 138 | 470 | 170 | 145 | 137 |
| Tran noise IRN [nVrms]  w/ electrode | 200 | 325 | 209 | 499 | 183 | 210 | 214 |
| Z_in_ (@50Hz) [MOhm] | 49.0 | 17.4 | 18.7 | 34.9 | 16.0 | 25.5 | 18.7 |
| Z_in_/Z_elec_ (@ 50Hz) [dBc] | 74 | 65 | 65 | 71 | 64 | 68 | 65 |

**S.2 CMRR, PSRR and Input Impedance**

The input impedance is measured using a 920kOhm resistor in series with the input of the AFE. The voltage drop over this resistor with a 100mVpp input was measured to extract the input impedance. The CMRR and PSRR are measured by applying a 100mVpp common-mode signal and a 100mVpp signal on the supply respectively. The differential signal measured at the output is divided by the input signal and by the in-band gain of the system to compute the CMRR and PSRR. The results are shown in Figure S1.


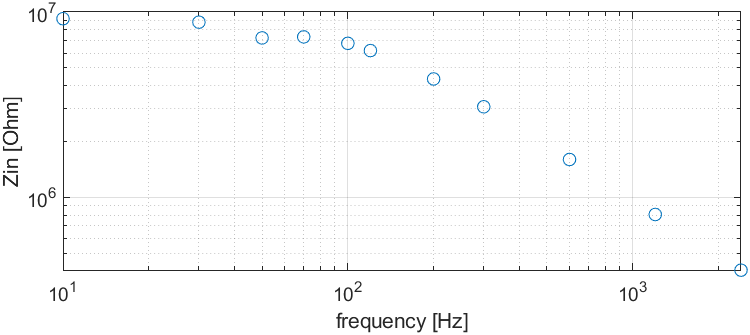

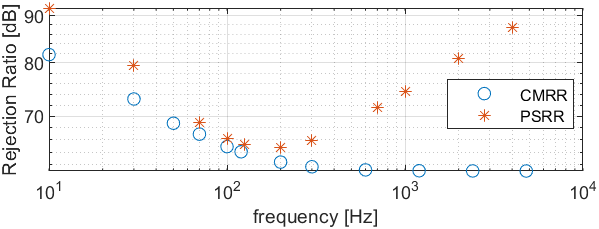


| (a) | (b) |
| --- | --- |

Figure S1: Measured (a) input impedance, (b) CMRR and PSRR over input frequencies from 10Hz to 5kHz.

**S.3 Electrode DC offset (EDO) measurements**

To test the sensitivity of the AFE to EDO, first, measurements are done with a differential EDO ranging from -150mV to 150mV and a differential input signal of 10mVpp, far above the maximum expected one. The gain and the THD (defined here as the sum of the 2^nd^, 3^rd^, 4^th^ and 5^th^ harmonic divided by the fundamental tone) are plotted in Figure S2.

Furthermore, measurements are done with a common-mode EDO of 0mV, +150mV and -150mV applied at the input. Figure S3(a) shows the transient behavior at the output with a 10mVpp differential signal applied to the input. A small offset between the different measurements appears at the output voltage due to the limited CMRR, however, the differential gain is unaffected. Figure S3(b) shows the input-referred noise with no signal applied at the input, but in presence of different EDOs (respectively 150mV, 0mV and -150mV). From the figure, it can be seen that the in-band noise is the same for all cases.


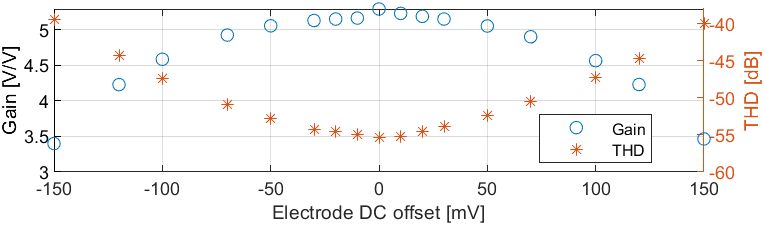


Figure S2: Measured gain and THD of the AFE with a 10mV differential input signal and an EDO between -150mV and 150mV applied at the input.


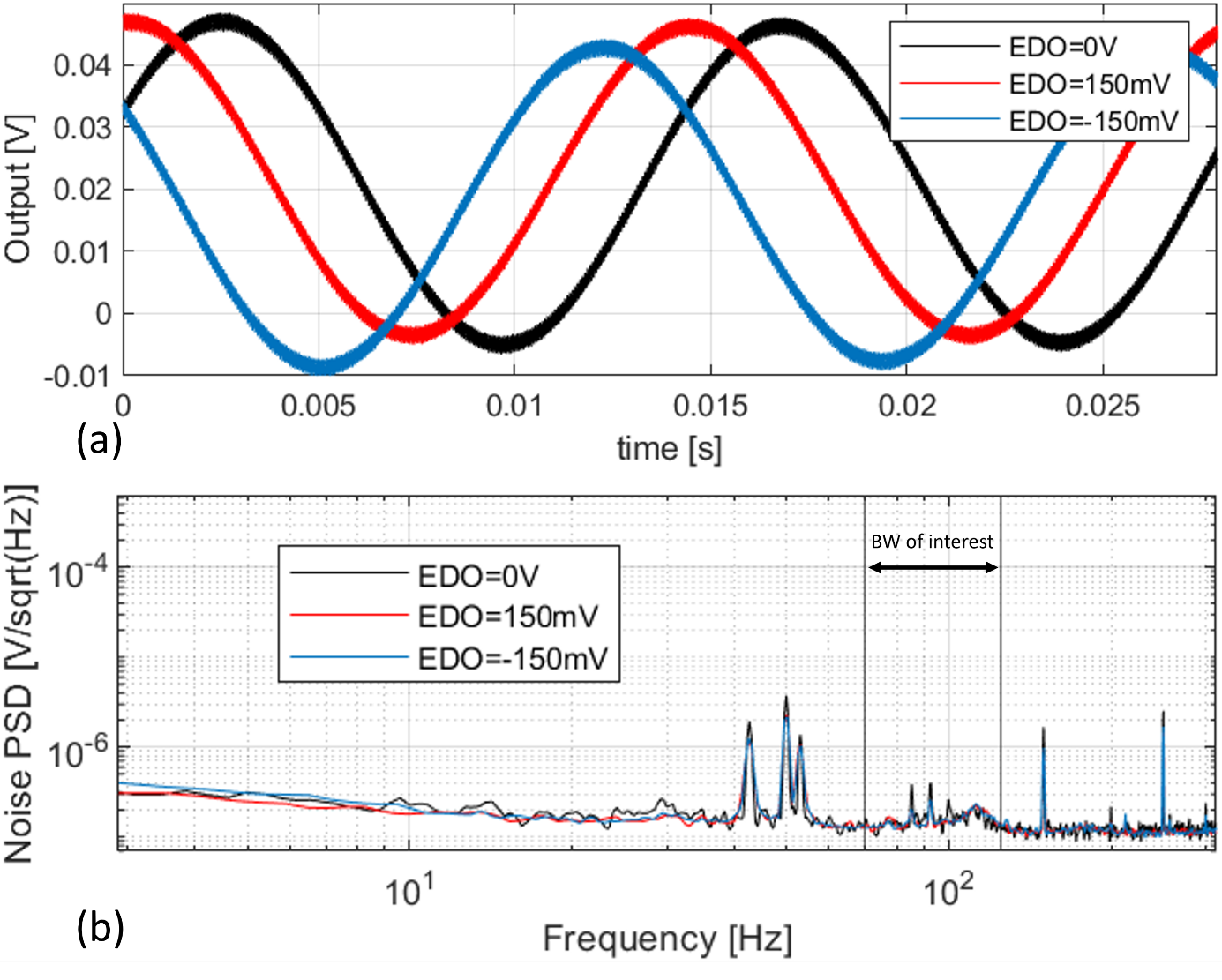


Figure S3: AFE characterization: measured (a) output differential voltage and (b) IRN at 0V, 150mV and -150mV common mode Electrode DC Offsets applied at the input.

**S.4 Electrode equivalent model measurements**

In order to simply evaluate the effects of the electrode impedance on the AFE performance, an approximated electrical model of the electrode intended to be used in a real application needs to be determined. To do so, impedance measurements are performed on the electrodes and the results shown in Figure S4 (magnitude and phase of the frequency response marked in blue). The electrode behavior is matched in the region of interest (70-125Hz) by using a linear RC network, featuring a capacitor C = 1µF in series with a resistor R = 1kΩ (red line in Figure S4).

To ensure a correct DC biasing of the AFE, a 1MΩ resistor is added in parallel to the capacitor C. By applying this equivalent network at the input of the AFE it is possible to characterize the impact of the electrode impedances on the circuit performance. In these conditions, the measured differential output voltage and extracted input-referred noise (IRN) are presented in Figure S5(a), (b) respectively. It can be seen that the AFE gain with (red) and without electrode emulating network (blue), are both about 5 V/V. Furthermore, the IRN level is also comparable in the bandwidth of interest. At relatively low frequencies, located outside the bandwidth of interest (<<70Hz), the IRN in Figure S5(b) reveals an increase in the floor due to the high-pass characteristics of the electrode. This is related to the biasing circuit used to supply the common mode voltage to the biasing resistor in the measurement setup.

**
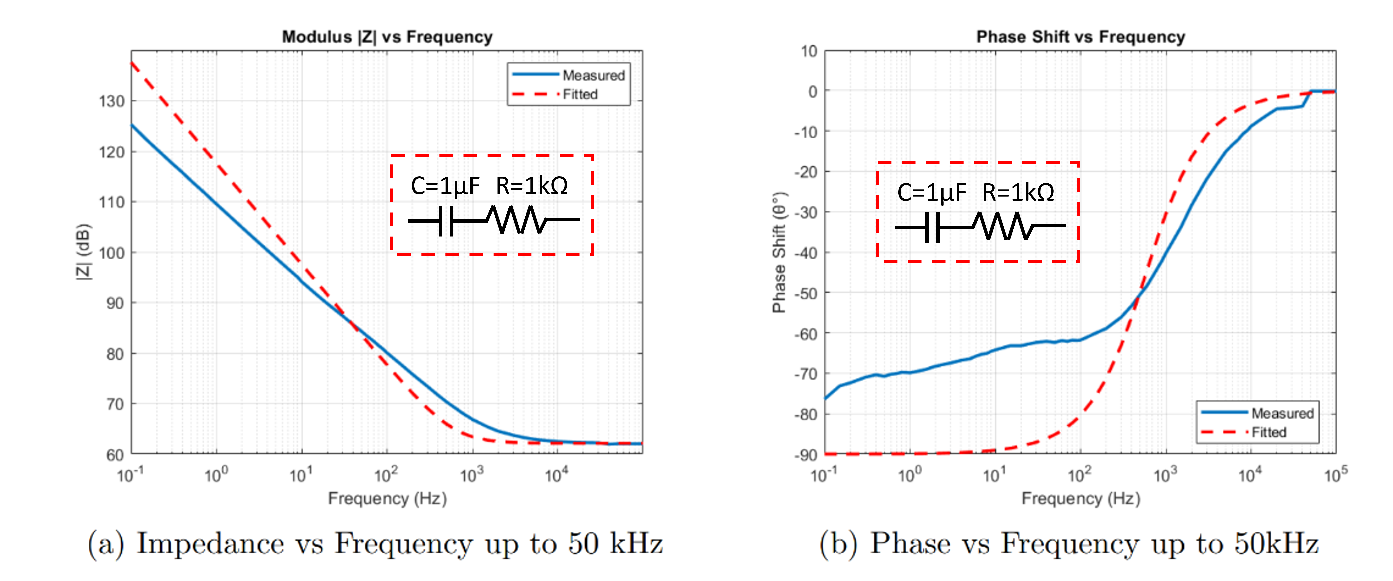
**

Figure S4: Impedance measurements of the electrode (blue) and fitted RC network behavior (dashed red) : (a) magnitude and (b) phase response.


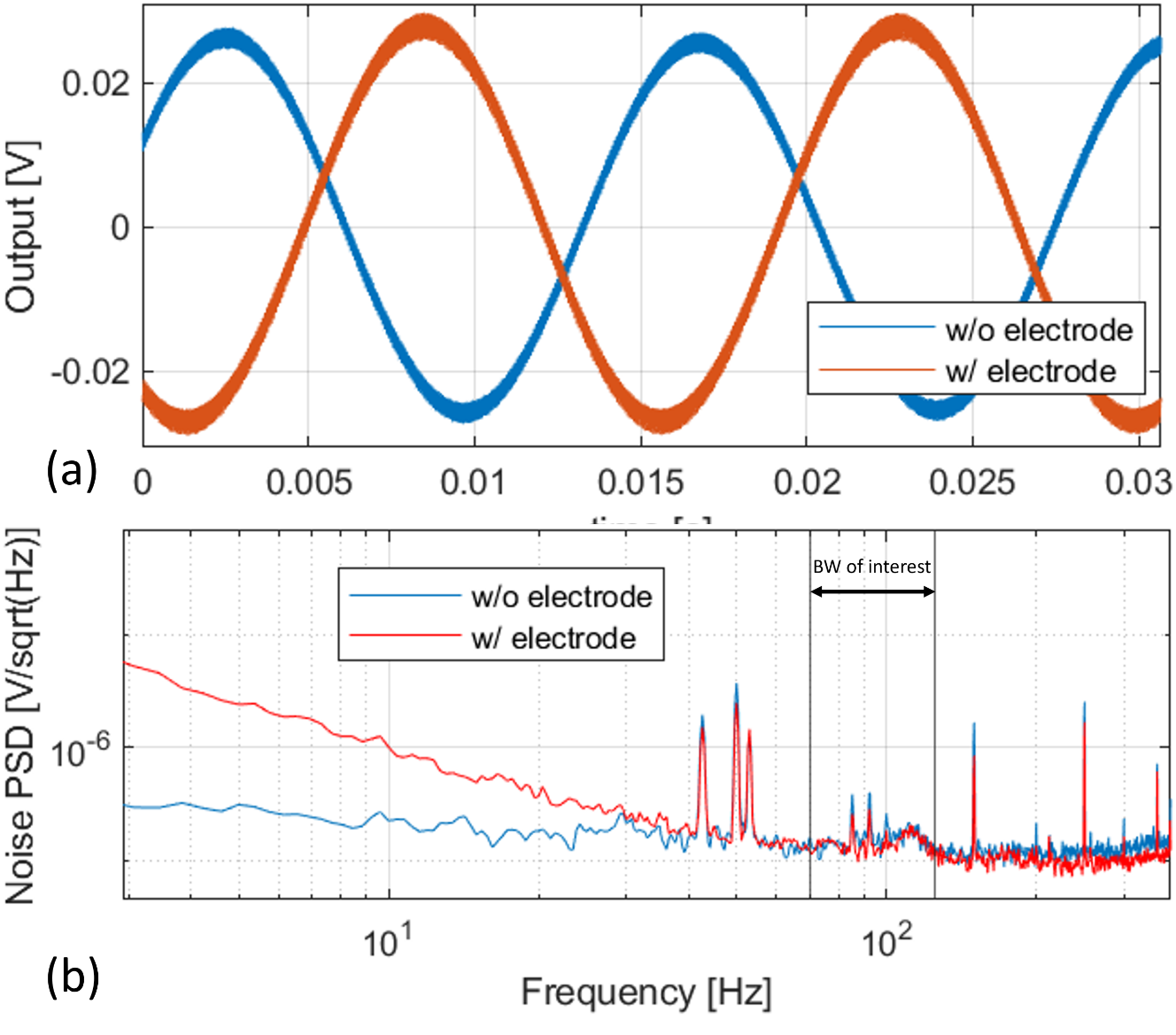


Figure S5: Measured (a) output transient signal and (b) input-referred noise (IRN) for a 10mVpp input, with (red) and without (blue) electrode model.
